# A database of antimalarial drug resistance

**DOI:** 10.1186/1475-2875-5-48

**Published:** 2006-06-15

**Authors:** Carol Hopkins Sibley, Pascal Ringwald

**Affiliations:** 1 Department of Genome Sciences, University of Washington, Seattle, WA 98195-7730, USA; 2 Global Malaria Programme, World Health Organization, 20 Av. Appia, 1211 Geneva 27, Switzerland

## Abstract

A large investment is required to develop, license and deploy a new antimalarial drug. Too often, that investment has been rapidly devalued by the selection of parasite populations resistant to the drug action. To understand the mechanisms of selection, detailed information on the patterns of drug use in a variety of environments, and the geographic and temporal patterns of resistance is needed. Currently, there is no publically-accessible central database that contains information on the levels of resistance to antimalaria drugs.

This paper outlines the resources that are available and the steps that might be taken to create a dynamic, open access database that would include current and historical data on clinical efficacy, in vitro responses and molecular markers related to drug resistance in *Plasmodium falciparum *and *Plasmodium vivax*. The goal is to include historical and current data on resistance to commonly used drugs, like chloroquine and sulfadoxine-pyrimethamine, and on the many combinations that are now being tested in different settings. The database will be accessible to all on the Web.

The information in such a database will inform optimal utilization of current drugs and sustain the longest possible therapeutic life of newly introduced drugs and combinations. The database will protect the valuable investment represented by the development and deployment of novel therapies for malaria.

## The problem

Whenever drug treatment is required to control a pathogen, selection of drug resistance is inevitable [1]. The huge size of pathogen populations and their short generation times guarantee the outcome. *Plasmodium falciparum *is a prime example. In humans, an acute infection can produce a population as high as 10^11 ^haploid parasites, so that mutations have ample scope to occur [2]. In the obligate sexual cycle, reassortment and recombination can "reshuffle the deck" for rapid evolution of new resistant genotypes in each generation. Thus, favoured combinations of genes can arise fairly quickly, even if more than one mutant gene is required for resistance. These sets of genes can, of course, also be separated during the sexual cycle but under the strong selection that drugs can exert, even a multigenic, resistant genotype may become fixed in a population.

The impact of this strong selection has been revealed at many different levels. Most important, as the use of chloroquine increased, drug resistance evolved in parasite populations and childhood mortality from malaria increased, even as all-cause mortality in children decreased [3-5]. The sequence of the *P. falciparum *genome has recently been published [6] and this has made it possible to trace the ancestry of highly drug-resistant parasites. These studies show that parasites resistant to chloroquine and sulfadoxine-pyrimethamine have arisen relatively rarely, but they have spread widely from a few initial foci in "selective sweeps" of the parasite population [7-11]. This new view affects many of the assumptions that underlie models of the speed at which resistance evolves [12] and inform practical decisions about changes in drug policy. Parasites without borders make it absolutely essential that the emergence of drug resistant populations be "tracked" worldwide; a resistant parasite that arises in Southeast Asia may travel rapidly to East Africa.

**Figure 1:**
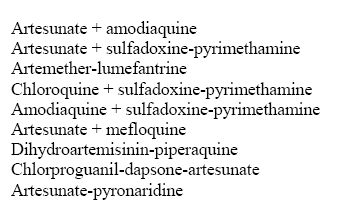
Drug combinations in use or in trials.

This improved understanding of the evolution of drug resistance has come from a relatively simple situation. Until recently, the number of antimalaria drugs in common use was small: chloroquine and sulfadoxine-pyrimethamine in Africa and the Americas, with mefloquine and more recently, mefloquine-artesunate in Southeast Asia[13]. As chloroquine and sulfadoxine-pyrimethamine have lost their efficacy, combination drugs have been strongly endorsed as the most effective next step [14]. In response to this emphasis, many different combination drugs, most containing an artemisinin derivative are being used in various countries, especially in East Africa (Figure 1. Many of these combinations have shown excellent initial efficacy in drug trials [13], but only mefloquine/artesunate has a long enough history to allow a strong prediction of the useful therapeutic life of these combinations [15]. It is particularly important to establish a baseline for effectiveness of new drugs and combinations so that any subsequent changes can be seen. This complex situation underlines the importance of regional surveillance of drug use, efficacy and effectiveness as these new combinations are tried in a variety of demographic and ecological settings. What has worked well for a long time in Thailand may not be so long lived in Tanzania [16]!

**Figure 2:**
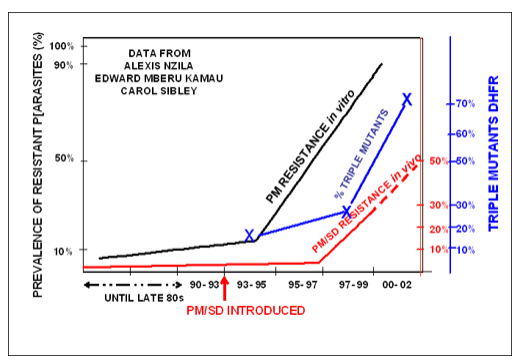
**Surveillance of efficacy of sulfadoxine-pyrimethamine in Kilifi, Kenya 1988–2002**. Pyrimethamine efficacy in vitro, prevalence of 51I/59R/108N alleles of *P. falciparum dhfr *and clinical efficacy of sulfadoxine-pyrimethamine in treatment of uncomplicated malaria in children were separately assayed in over the indicated period using standard assays [52, 69, 70]. The data are used with permission of Dr. Alexis Nzila and Dr. Edward Mberu Kamau.

Appropriately, the gold standard for drug efficacy has been the outcome of clinical treatment. When patients are treated with the drug, do they recover? The substantial expense and logistical difficulty to change the recommended drug treatment have led most countries to rely on a large increase in clinical treatment failure before initiating a change [17]. Systematic studies have shown repeatedly that assessment in vitro of drug efficacy in local parasite isolates can give an early warning of rising drug resistance in vivo [18-20]. In addition, when molecular correlates of drug resistance are known, the prevalence of resistant alleles can also give early warning of evolving resistance in the parasite population [21-23]. In all three approaches, the temporal and geographic patterns of resistance are most informative. When the in vitro tolerance of parasites to a drug is rising or when the prevalence or the geographic range of resistant alleles is increasing, clinical drug failure is likely to increase as well. Figure 2 shows a small example of the linkage among the three parameters. In this data set, the increase in the in vitro IC_50 _values and the increased prevalence of the triple mutant allele of *P. falciparum dhfr *preceded by several years the increase in sulfadoxine-pyrimethamine treatment failure among young children in Coastal Kenya. Similar studies will be needed to determine whether the lags between these parameters observed in Kilifi will be similar in other sites or for other drugs, but it is clear that the in vitro increase in IC_50 _values and the increase in the molecular marker can provide an early warning of the onset of clinical treatment failure. The community will need similar data sets in many different settings for all of the drugs in use to manage effectively the current drugs and any novel drugs that are introduced in the future.

## A solution: a real-time public database

A large amount of data of all three kinds – in vivo patient response to treatment, in vitro response of patient isolates to drugs and prevalence of molecular markers of drug resistance – is currently being collected in malaria-endemic areas. Unfortunately, much of this information is sitting unused and unusable in journals, meeting abstracts, Ministry of Health reports and other informal sites. An open, public database would record the data in real time so that the data could be accessed, analysed and productively used by all interested parties.

## What resources are currently available?

There are already many sources of historical data that could be incorporated readily into such a database. First, WHO has just published a report on the "Susceptibility of *Plasmodium falciparum *to antimalarial drugs. A report on global monitoring 1996–2004". This report gathers the available published and unpublished information on the tools used in monitoring drug efficacy, the data from endemic countries and the trends observed in the monitoring period [13]. The report, underpinned by a web-based global database, serves as a meta-analysis of the current situation, but it does not provide access to the original data. This WHO database serves primarily the purpose of documenting the evidence for treatment policy change in malaria endemic countries. It is updated every three months and includes analysis of trends in the data. The nature and the country ownership of these data available to WHO do not lend themselves to direct deposition in a universal database. However, sharing of these data for specific and agreed purposes is possible. In this way, the information in the WHO database could form the core of an open database.

A second valuable source is the national surveillance programmes that have comprehensive data sets. For example, the IMPACT-Tz programme, (Interdisciplinary Monitoring Project for Antimalarial Combination Therapy in Tanzania, [24] is a joint programme of the United States Centers for Disease Control and the Ifakara Health Research and Development Centre and includes partners at the National Institute for Medical Research and Muhimbili University College of Health Sciences, Dar-es-Salaam, Tanzania, London School of Hygiene and Tropical Medicine and the Swiss Tropical Institute. This study focuses on the effectiveness of artemisinin-based combination therapy in areas with intense malaria transmission. Similar programmes with many collaborators have been launched in other countries and the inclusion of such national data sets would be extremely valuable.

**Figure 3:**
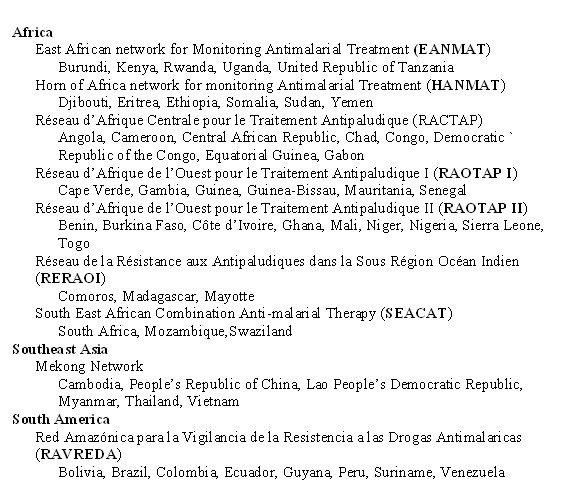
Regional surveillance networks.

**Figure 4:**
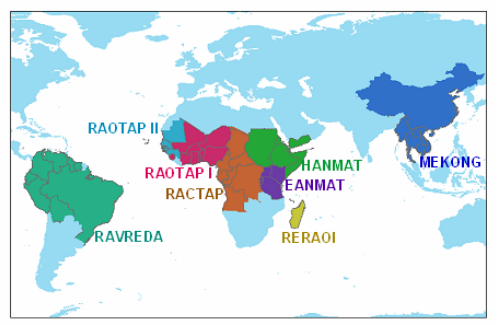
Some regional networks for surveillance of antimalarial drug efficacy.

Third, several networks for regional monitoring of current trends have been established. The prototype is EANMAT- the East African Network for Monitoring Antimalarial Treatment. This is a regional network that established common protocols to monitor the efficacy of the first and second line drugs in each national control programme [25,26]. The data are collected annually and reported on a common website [27]. Similar networks have been established in other regions of Africa, Southeast Asia and South America (Figures 3, 4). Collection of data from these network web sites could be an important contribution to a comprehensive international database. Figure 4 shows a map of some regional networks that have been organized so far.

Fourth, there are supra-national networks that are collecting and archiving data on parasite response to drugs and on polymorphisms related to resistance. For example, the Pasteur Institutes in Senegal, French Guyana, Madagascar, Cambodia and Ivory Coast are combining field studies, molecular biology and in vitro monitoring of drug responses in parasite populations to identify early signs of declining drug efficacy [28]. The US Department of Defense also has a comprehensive network collecting data on various emerging infectious diseases Global Emerging Infections System: [29], and malaria is included in their portfolio. The WHO and the Multilateral Initiative Against Malaria have also formed a network in five African countries [30]. The varied environments represented by these centres, their common protocols and their integrated approach are exactly the sort of input one would want to include in a common database.

Fifth, there is a rich archive of data on the mechanisms of action, interactions between drugs and the speed with which resistance to antimalarial drugs can be selected in rodent models [1,31-34]. Inclusion of these data in the central archive can also provide information on the genetic basis for resistance to particular drugs, information that can then be compared with data gathered in human parasites.

Finally, there is a growing archive of molecular data on the prevalence of alleles implicated in resistance to chloroquine, sulfadoxine-pyrimethamine, mefloquine, quinine and artemisinin related compounds [35]. Much of this information is contained in publications and meeting abstracts and its usefulness is often limited because the reported data derive from a limited area or capture the information from a single time point. Like the in vitro assessments, the molecular data do not relate directly to clinical outcomes in individual patients; too many important host factors influence the clinical outcome. The utility of the molecular markers lies in the overview of the geographic and temporal trends that can easily be discerned from coordinated sets of the data [21-23,36,37]. For example, alleles of *P. falciparum Pfcrt *that carry the key K76T mutation are strongly associated with resistance to chloroquine and the prevalence of that allele declined dramatically in Malawi after chloroquine was replaced with sulfadoxine-pyrimethamine as the first line drug in 1993 [38]. This decline was paralleled by a return of the clinical efficacy of chloroquine in Malawi. Similar associations of molecular markers for sulfadoxine-pyrimethamine and mefloquine have been defined [21,39,40], and recent data have identified markers that may be useful in tracking quinine and artemisinin resistance as well [40-42]. More work will be needed to determine the clinical significance of some of these newly identified molecular changes. When these correlations have been made, similar monitoring of baseline levels and any changes in allelic prevalence could follow the evolution of resistance to other drugs as they are introduced. The increase in particular alleles could serve as an early warning as resistant parasites gain ground in an area. As with the monitoring of in vitro drug sensitivity, there is a lag between the rising prevalence of a resistant allele and the increase observed in clinical failure and that lag period will need to be determined for each location.

The ease of the molecular studies, the fact that the analysis requires only a few drops of blood and the stability of dried samples on simple filter papers makes this an attractive approach. In fact, several of the regional networks are routinely archiving these dried blood samples, so that molecular studies will be easy to realize, even if the samples are not analysed immediately. For many of the new drugs and combinations, molecular correlates are yet to be defined. In these cases, archives of dried samples could be used retrospectively (with appropriate patient consent) to define baseline data on allelic prevalences from periods before the drugs were even introduced. This would be an extremely valuable dataset from which to gauge temporal changes after deployment of new drugs and combinations Many of the national and regional programmes are archiving samples in this way, and numerous laboratories in endemic countries have the facilities and expertise to analyse the samples for the relevant genetic changes. As data of this kind are published, collation of the molecular markers will allow users to query the database to follow geographic and temporal trends in real time [43].

## How would a database work?

The format of such a database will require careful planning by the whole malaria community. The sources, such as those cited above, could provide three complementary types of data:patient responses to drugs, in vitro efficacy of drugs in use, or those that might be adopted, and molecular markers correlated with resistance to drugs. The clinical data will be the most complex and varied, and it seems sensible to create separate sections of the database with easy linkages among the three parts. The three approaches provide complementary information and each of them will need to be integrated into the overall database.

The first step is to decide what data are to be included and how these are to accessed. Although this review focuses on *P. falciparum*, there are also data on *P. vivax *clinical efficacy and some data on molecular correlates of resistance, as well. These should also be included [44,45]. A meeting of potential contributors and end-users would be a sensible way to initiate the discussion. At the outset, published information can be collected, and then added to the base as new information becomes available, as is currently done with genomic and protein structure data. As noted above, there are significant data in smaller databases already and, with permission, these could be incorporated reasonably readily into the central core, with links to each individual database. There are some models that may be useful. For example, there is a very impressive HIV site that includes a wide range of information, including drug effectiveness, resistant mutants and the relationship to patient outcomes after treatment [46].

The many "omics" databases that have been created in recent years have given considerable experience in managing and integrating large volumes of data. At the outset, it will be critical to reach a consensus on standardized input and output. Such a database will be useful only if it is maintained, so the input format must be accessible and straightforward. Moreover, the output must be useful to a wide variety of users, from individual scientists and health care professionals to those charged with making public health decisions. Simplicity of input, user friendly interfaces and broad utility of the output are not easy to attain, and the creation of the database will require sophistication. However, this task can only begin when the community has decided on input and output.

Most people envisage maps as one useful output. The MARA project that integrated climate data with demographic information and malaria incidence showed clearly the power of a visual presentation [47-49]. Similarly, WHO/GMP has informative maps for many individual African countries [50]. The capacity to view resistance on both continent wide and regional bases and to examine changes in these parameters over time would greatly enhance our understanding of the evolution of resistance.

## What are the anticipated problems?

### Technical issues

Assembling the data will not be an easy task and clinical data present the biggest challenge. Even the data in organized trials of drug efficacy are heterogeneous, and when more information from routine surveillance is included, the problem is compounded. Although WHO standard protocols have been published since 1996, the inclusion criteria, protocols used and end points measured in particular studies frequently diverge based on the needs and constraints of each particular study. It has been recognized for a long time that the approaches appropriate for areas of very high transmission are not sensible for those where malaria is rare [51,52]. The past controversy over whether patients should be followed for only 14 days after treatment to avoid categorizing new infections as recrudescence, or for 28 days or more to observe late return of the parasites is a good example. Even when one attempts to use molecular markers to distinguish new infections from those that have recrudesced, many difficulties still remain [53].

These complexities are not unique to malaria; they occur whenever clinical assessments need to be made. Meta-analyses like those done in the Cochrane Systematic Reviews [54] are now routinely used to compare protocols and outcomes in a wide variety of clinical situations to identify the best practice based on hard evidence. In fact, several analyses of antimalaria drug efficacy have been published in the Cochrane series in the last 2 years [55-58]. A comprehensive database for the much more difficult and contentious problem of all clinical trials has also been recently proposed [59]. A major issue for antimalarial drug resistance is that most of the historical data on drug efficacy were collected under the 1996 WHO recommendation for 14 day follow-up, but many studies have now shown that this short protocol markedly underestimates true drug failure, especially for long-lived drugs [60]. The WHO guidelines were revised in 2001 to recommend at least 28 days of follow-up. This presents a dilemma; exclusion of these earlier data would ignore a huge amount of extremely valuable information. After all, clinical treatment failure before 14 days is a very important finding, even if apparent adequate clinical response within that time often masks parasites that recrudesce within 28 days [60]. Despite these complexities, retrospective analyses by White and his colleagues [60,61] have been used productively to draw common conclusions from the many clinical trials that have been conducted.

A consensus on the quality controls, endpoints, inclusion criteria and sampling protocols used in each study is a prerequisite. Then, detailed information on each study can be entered in the database with a controlled vocabulary. Under this system, an individual user can decide whether to include studies with a 14 day follow-up in the analysis and query the database with that stipulation as one argument. As long as the 14-day studies can easily be identified in searches of the database, an individual can access and analyse the data with that limitation in mind.

Assessment of drug sensitivity in vitro avoids many of the host factors that complicate in vivo studies and this approach makes it possible to determine the level of resistance to individual drugs. With the widespread adoption of combination therapies, this kind of assessment will be even more important for following trends in resistance to the components of the various combinations. It is often impossible to conduct therapeutic efficacy tests for each component, owing to ethical problems, non-availability of the drug as a single therapy and the need to study a large number of patients. In vitro tests can be used to monitor susceptibility to each drug in a combination. This is especially important in the current situation where many different partners are being paired with artemisinin derivatives. Failure of the partner may be more likely than resistance to the artemisinin component, but resistance to the artemisinins would compromise virtually all of the combinations currently in use or development. At the moment, in vitro efficacy studies are the only tool available to track this key parameter.

There are numerous difficulties in comparing the absolute IC_50 _values derived in different laboratories. It is particularly important to distinguish studies that assess contemporary patient isolates, rather than those limited to culture-adapted reference strains. Fresh patient isolates may contain more than one strain, but they are important because they do reflect the current situation. In addition, other parameters can differ from one laboratory to another, including the media used, the protocols for assessment of parasite growth and the reference strains assessed [62,63]. Again, discussion of these differences will be needed in order to define parameters that will allow comparison of data collected in different laboratories. For example, the most valuable information may be the temporal trend in values measured with a consistent protocol in a particular laboratory, especially if those values compare the response of freshly isolated parasites with standard reference strains.

The inclusion of molecular data on alleles correlated with resistance to a particular drug presents fewer complexities. There are various protocols for the determination of alleles and the sensitivity of the protocols to minor alleles in the isolates from patients with polyclonal infections varies enormously [64-67]. Despite these differences, the overall assessment of the prevalence of resistant alleles in a parasite population is fairly consistent among the various approaches.

Molecular analysis is also used to distinguish recrudescence from new infections in patients in whom parasites return later than 14 days after treatment. The alleles of very polymorphic genes like *msp1*, *msp2 *and *GLURP *are often used, and the sequence of the whole genome now allows microsatellite or single nucleotide polymorphisms to be used as markers. Both the sensitivity of the detection used and the definition of a new or old parasite have a profound influence on the assessment of success or of drug failure [53], so a common definition of these issues is needed if data from clinical different studies are to be compared.

### Human and programmatic issues

Many human factors will also need to be resolved if such a database is to be created. Gathering data with all of these methods is a complex, demanding activity that requires a high level of professional training and dedication. Rapid release of these data to a database before publication would deprive the scientist of professional recognition for the hard work involved in data collection. Again, this is not unique to the malaria community; the genome and public health surveillance networks have faced similar problems. At the outset, this difficulty can be mitigated by establishing the database with published data. Ultimately, the community will need to devise ways of recognizing and rewarding the important effort of surveillance. Without this recognition, the motivation to collect high quality data may be low and inclusion of inaccurate data serves no one.

The use of human samples raises important issues of consent and patient privacy, even when only the parasite parameters are evaluated. If molecular or in vitro analysis or publication of patient data in a database is anticipated, the appropriate informed consent can be sought from the patient or guardian, at the time of the study. However, for archival samples, this is usually impossible. These issues will need to be carefully addressed for each data set that is considered for inclusion in a database that is publicly available. Again, many of these issues have been addressed in other databases and the needed safeguards can be built into the database. A recent paper from EM Kamau addresses thoughtfully many of these issues [68].

## Conclusion

The bottom line is clear: continuing to treat patients with failed or failing antimalarial drugs is a major and unnecessary cause of mortality. The introduction of artemisinin-based combinations may reverse that trend, but resistance to these drugs will evolve eventually. WHO now recommends that review and change of the antimalarial treatment policy should be initiated when the cure rate with the current recommended medicine falls below 90% (as assessed in the course of surveillance monitoring) and the new recommended treatment should have an average cure rate of ≥95% as assessed in clinical trials. It is crucial to establish and maintain close surveillance as new drugs are introduced so that they will have the maximum useful therapeutic life. The first step is to assemble and integrate the information now available, and assemble a comprehensive real time database that is accessible to all. Postponing this synthesis will only make it harder to integrate the current information. It is imperative to act now to make the community decisions that will initiate this effort.

## Authors' contributions

CHS and PR wrote the paper jointly.
